# Individual volume‐based 3D gamma indices for pretreatment VMAT QA

**DOI:** 10.1002/acm2.12062

**Published:** 2017-03-20

**Authors:** Jinling Yi, Ce Han, Xiaomin Zheng, Yongqiang Zhou, Zhenxiang Deng, Congying Xie, Xiance Jin, Fu Jin

**Affiliations:** ^1^ Department of Radiotherapy and Chemotherapy The 1st Affiliated Hospital of Wenzhou Medical University Wenzhou China; ^2^ Physics Unit Department of Radiation Oncology Chongqing Cancer Hospital & Institute Chongqing China

**Keywords:** percentage dosimetric errors, percentage gamma passing rate, quality assurance, volumetric‐modulated arc therapy

## Abstract

Although gamma analysis is still a widely accepted quantitative tool to analyze and report patient‐specific QA for intensity‐modulated radiotherapy (IMRT) and volumetric‐modulated arc therapy (VMAT), the correlation between the 2D percentage gamma passing rate (%GP), and the clinical dosimetric difference for IMRT and VMAT has been questioned. The purpose of this study was to investigate the feasibility of individual volume‐based 3D gamma indices for pretreatment VMAT QA. Percentage dosimetric errors (%DE) of dose‐volume histogram metrics (includes target volumes and organ at risks) between the treatment planning system and QA‐reconstructed dose distribution, %GPs for individual volume and global gamma indices, as well their correlations and sensitivities were investigated for one‐ and two‐arc VMAT plans. The %GPs of individual volumes had a higher percent of correlation with individual 15 %DE metrics compared with global %GPs. For two‐arc VMAT at 2%/2 mm, 3%/3 mm, and 4%/4 mm criteria, individual volume %GPs were correlated with 9, 12, and 9 out of 15 %DE metrics, while global %GPs were correlated with only 2 out of 15 %DE metrics, respectively. For one‐arc VMAT at 2%/2 mm, 3%/3 mm, and 4%/4 mm criteria, individual volume %GPs were correlated with 18, 16, and 13 out of 23 %DE metrics, and global %GPs were correlated with 19, 12, and 1 out 23 %DE metrics, respectively. The area under curves (AUC) of individual volume %GPs were higher than those of global %GPs for two‐arc VMAT plans, but with mixed results for one‐arc VMAT plans. In a conclusion, the idea of individual volume %GP was created and investigated to better serve for VMAT QA and individual volume‐based %GP had a higher percent of correlation with DVH 15 %DE metrics compared with global %GP for both one‐ and two‐arc VMAT plans.

## 1 Introduction

Volumetric‐modulated arc therapy (VMAT) is a novel delivery method of intensity‐modulated radiotherapy (IMRT). It is capable of delivering highly conformal dose distribution through simultaneous continuous gantry rotation, dynamic beam modulation, and variable dose rate.^1^, ^2^ Due to its rotational delivery feature and increased complexity in planning and delivery, there has been increased emphasis on the need for attention to safety and quality assurance (QA) processes involved in VMAT treatment.^3^, ^4^

Although *γ*‐analysis is still a widely accepted quantitative tool to analyze and report patient‐specific QA for IMRT and VMAT, the correlation between the 2D percentage gamma passing rate (%GP) and the clinical dosimetric difference for IMRT and VMAT has been questioned.^5^, ^6^ Since the general 2D %GP tells only how many voxels fail to pass the criteria and provides no information on the anatomic location of the failure or at which dose level it failed, dose‐volume histogram (DVH)‐based metrics relied on 3D dose reconstruction has been suggested to replace the 2D %GP for IMRT and VMAT QA.^6^, ^7^ 3D *γ*‐analysis was introduced and applied to compare the treatment planning system (TPS)‐computed dose distribution and measurement‐reconstructed 3D dose distribution for IMRT QA purpose.^8^, ^9^

Currently, several QA methods are available for 3D dose reconstruction based on measurement, such as LINAC on‐board detectors,^10^ diode array,^11^ EPID panels,^12^ and LINAC control system log files.^13^ However, the studies on 3D *γ* indices, especially individual volume‐based *γ* indices and their correlation with clinical dosimetric differences were limited. The purpose of this study was to investigate the effective individual volume‐based 3D *γ* indices for VMAT QA based on 3D‐reconstructed dose distribution and their correlation with dosimetric differences in both model‐based and measurement‐based dose reconstructions.

## 2 Materials and methods

### 2.A Study design

**Figure 1:**
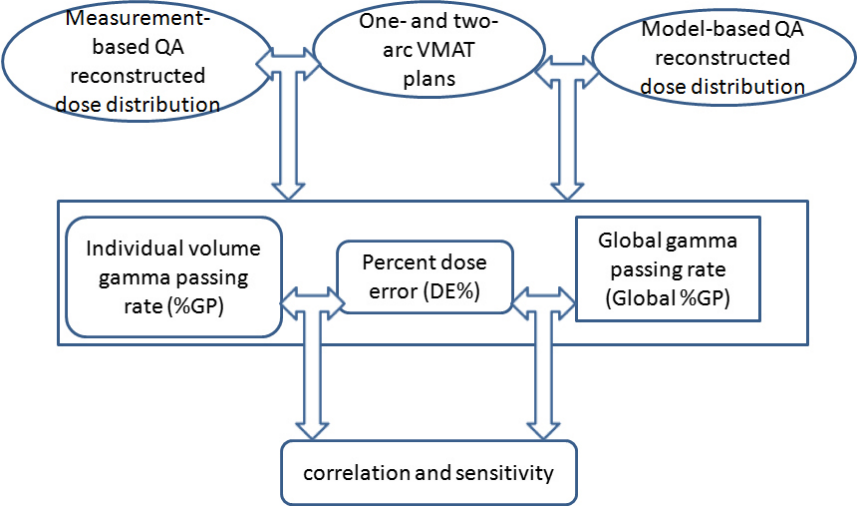
Flowchart for the whole study design.

As shown in Fig. 1 the flowchart for the overall study design, One‐ and two‐arc VMAT plans were verified with model‐based and measurement‐based QA to acquire the percent dose errors (%DE) between planed and QA‐reconstructed dose distributions, as well as the global gamma passing rate (%GP) and individual volume‐based gamma passing rate (%GP). Statistical correlations between %GP and %DE were investigated using Pearson's correlation coefficient. The sensitivities of individual volume‐based %GP and global %GP were then investigated and compared.

### 2.B Patients and planning

Thirty‐one nasopharyngeal cancer (NPC) patients who underwent two‐arc VMAT treatment and 33 prostate cancer patients who underwent one‐arc VMAT treatment were enrolled in this study. VMAT plans were optimized with the SmartArc algorithm in Pinnacle treatment planning system (TPS) (Philips Healthcare, Fitchburg, WI) for a 6‐MV photon beam on an Elekta Synergy^®^ linac (Elekta Ltd, Crawley, UK) equipped with an 80‐leaf MLC (MLCi2™, Elekta Ltd, Crawley, UK). Two‐arc VMAT objective settings and optimization parameters for NPC patients have been reported in previous studies.^14^, ^15^ Briefly, prescription doses were 70 Gy and 56 Gy for gross tumor volume (GTV) and planning target volume (PTV) in 28 fractions, respectively. Organ at risks (OARs) consisting of the brainstem, spinal cord, left and right parotids, as well as lens were constrained for optimization. The first arc rotated clockwise from 181° to 180°, and the second arc rotated counterclockwise from 180° to 181°.

For one‐arc VMAT plan of prostate cancer patients, target delineation was done by one radiation oncologist according to the contouring guidelines of Radiation Therapy Oncology Group (RTOG) Trial 0126.^16^ GTV encompassed the prostate gland, CTV encompassed GTV plus the proximal bilateral seminal vesicles. PTV was generated by adding a surrounding margin of 7 mm to CTV. A total of 78 Gy dose was prescribed to PTV at 39 fractions. OARs were outlined according to the Male RTOG Normal Pelvis Atlas.^17^ For one‐arc VMAT optimization, at least 95% of PTV must be covered by 95% of the prescription dose. OAR constraints included rectum, bladder, peritoneal cavity or bowel, femoral heads, and unspecified tissue. A start angle of 181° and a stop angle of 180° were applied for one‐arc plans using clockwise (CW) rotation direction. A leaf motion of 0.46 cm/deg and a final arc space degree of 4 were employed for both one‐arc and two‐arc VMAT.

### 2.C Model‐based QA and measurement‐based QA

In this study, model‐based and measurement‐based QA were conducted with COMPASS system (version 1.2, IBA Dosimetry, Schwarzenbruck, Germany),^18^ which includes an two‐dimensional ion chamber (IC) array (MatriXX, IBA Dosimetry) and dose reconstruction software based on a beam model describing the characteristics of the accelerator (e.g., energy spectrum, lateral beam quality variations) and a collapsed‐cone convolution/superposition (CCC/S) algorithm.^19^ A strict commissioning of the whole system, including the validation of accuracy for 2D‐IC array measurement, beam modeling, and dose reconstruction, was performed in advance according to the same standards as the clinic used TPS.^20^

A model‐based dose reconstruction was performed by importing all the DICOM files (RT plan, RT dose, RT structures, and computed tomography [CT] images) of patients from Pinnacle into COMPASS. A 3D dose deposition on the patients’ CT dataset was reconstructed without measurement using CCC/S algorithm based on the commissioned fluence model and the dose engine to provide an independent dose verification for TPS calculation.

A measurement‐based QA was conducted by using MatriXX IC array with 5 cm RW3 (water‐equivalent phantom) (PTW, Germany) buildup slabs and gantry angle sensor placed on a gantry holder mount. VMAT plans were firstly delivered to MatriXX and the dose–response was measured. Predicted dose–response was calculated by COMPASS system using DICOM RT plan parameters (gantry angle, MLC position, MU, etc.) from TPS, detector model, and inbuilt beam model. The final 3D dose distribution on patient CT was reconstructed based on MatriXX measurement taking the difference between predicted dose–response and the measured dose–response into account. During dose reconstruction, the effective resolution was increased from 1 cm to 2 mm by a fit procedure to best fit the response of measurements.^20^ All plans were delivered through a record and verify system (MosaiQ^®^ v. 1.60Q3, IMPAC Medical Systems, Inc., Sunnyvale, CA, USA).

### 2.D DVH‐based metrics dose evaluation and 3D *γ‐*analysis

Percentage dosimetric errors (%DE) of DVH metrics between the TPS and the model‐based and measurement‐based QA‐reconstructed dose distribution were recorded and compared. %DE for each metric was defined as (Dcompass‐D_TPS_)/D_TPS_×100. For target coverage, Dmean, D2, and D98 (dose to 2% and 98% volume), V95 (percent volume irradiated by 95% of the prescription dose) of GTV and PTV were calculated and compared. For OARs of NPC patients, D1 (dose to 1% volume) of the spinal cord, brainstem, and lens, Dmean and D50 of parotids were evaluated. For prostate cancer patients, the Dmean, D40, and D70 (dose delivered to 40% and 70% volume) of bladder, Dmean, D5, V50, V60, V70 of rectum, Dmean and D2 of left and right femoral heads were calculated. All patient plans were calculated with a dose grid of 2 mm × 2 mm × 2 mm.

Relative percentage gamma passing rate (%GP) for individual target and OAR volumes, defined as individual volume‐based gamma index, such as *γ*GTV, *γ*PTV, *γ*brainstem, *γ*bladder, etc., were calculated with three different acceptance criteria: 4%/4 mm, 3%/3 mm, 2%/2 mm, respectively, with 10% lower dose threshold (TH). Relative global %GP, defined as the gamma pass rate for the whole patients during QA analysis were also calculated with three different acceptance criteria: 4%/4 mm, 3%/3 mm, 2%/2 mm, respectively, and 10% lower dose threshold (TH).

### 2.E Correlation and sensitivity analysis

Statistical correlation between 3D %GP of individual volume and %DE, as well as correlation between global %GP and %DE were investigated using Pearson's correlation coefficient (r) with SPSS 17.0 (spss Inc., Chicago, IL, USA). %DE was assumed to be correlated with a determined %GP when *P* < 0.05, which was obtained from r. In order to compare the sensitivities of 3D %GP of individual volume and global %GP, the number of “false negative” (FN) cases (cases with high QA passing rates but with large errors in DVH dose metrics) and “true positive” (TP) cases (cases with low QA passing rates and with large errors in DVH dose metrics) were calculated. In particular, we considered all those structures “FN” that had DVH metrics errors > 3% among those patients with %GP > 95%. We considered all the cases “TP” that had DVH metrics errors > 3% and %GP < 95%. From the FN and TP rates, receiver operating characteristic (ROC) curves were generated to investigate the ability of individual volume %GP and global %GP to identify accurately the plan with dose errors > 3%.^6^

## 3 Results

Table 1 shows the average %DE on different DVH metrics between TPS and model‐based, measurement‐based dose reconstruction from COMPASS for NPC and prostate cancer patients, respectively. Most of %DE of the DVH metrics were within 3%. However, higher dose differences were observed on percentage volume of certain isodose line, such as V95 of GTV for NPC patients, V95 of PTV, V60, V70 of bladder and rectum, and V50 of rectum for prostate cancer patients. D1 of lens, which has a small volume, was also presented with a relatively higher dose difference.

**Table 1:** Percentage dose differences between TPS and model‐based reconstructed dose, measurement‐based reconstructed dose for NPC and prostate cancer patients

	NPC		Prostate		
	Model based	Measurement based	Model based	Measurement based	
GTV					GTV
D2	1.15 ± 1.18	0.43 ± 1.08	−0.52 ± 0.66	−1.10 ± 0.84	D2
D98	−1.57 ± 1.27	−1.37 ± 1.0	−1.67 ± 1.02	−2.14 ± 0.86	D98
Dmean	−0.38 ± 0.74	−0.58 ± 0.63	−0.87 ± 0.73	−1.22 ± 0.68	Dmean
V95	−4.95 ± 4.25	−3.81 ± 3.01	−1.32 ± 4.72	−2.91 ± 3.23	V95
PTV					PTV
D2	0.36 ± 0.93	−0.13 ± 0.82	−0.55 ± 0.65	−1.10 ± 0.76	D2
D98	0.28 ± 2.24	−0.11 ± 2.18	−2.58 ± 1.17	−3.29 ± 1.08	D98
Dmean	0.35 ± 0.74	−0.09 ± 0.64	−1.06 ± 0.75	−1.42 ± 0.65	Dmean
V95	−1.56 ± 2.73	−1.55 ± 1.71	−5.34 ± 3.89	−6.89 ± 4.11	V95
Brainstem					Left femoral head
D1	−2.98 ± 1.99	−3.03 ± 2.42	−1.09 ± 1.39	−2.65 ± 1.47	Dmean
Cord D1	−0.75 ± 1.67	1.44 ± 3.50	−1.21 ± 1.67	−0.85 ± 3.06	D2
Left parotid					Right femoral head
Dmean	−0.98 ± 1.59	−0.65 ± 2.48	−1.57 ± 1.1.5	−2.35 ± 1.93	Dmean
D50	−0.96 ± 2.98	1.57 ± 4.70	−1.79 ± 1.27	−1.77 ± 2.09	D2
Right parotid					Bladder
Dmean	−2.61 ± 2.05	−3.45 ± 3.55	−0.52 ± 1.24	−1.78 ± 1.1.5	Dmean
D50	−2.41 ± 3.43	−2.35 ± 4.62	0.06 ± 2.68	−1.88 ± 2.34	D40
Lens D1	15.26 ± 11.34	13.07 ± 10.69	1.08 ± 6.23	−6.05 ± 5.83	D70
			−0.32 ± 1.51	−1.01 ± 1.36	V40
			−4.12 ± 3.11	−4.10 ± 4.29	V60
			−30.00 ± 36.38	−38.88 ± 32.84	V70
					Rectum
			−2.91 ± 1.71	−4.68 ± 1.61	Dmean
			−2.82 ± 1.90	−3.38 ± 1.69	D5
			−8.95 ± 5.23	−12.30 ± 4.85	V50
			−19.06 ± 9.58	−25.12 ± 10.09	V60
			−59.36 ± 44.43	−67.88 ± 36.42	V70

The %GPs of individual volumes and global gamma of NPC and prostate cancer patients with different criteria were shown in Table 2 for both model‐based and measurement‐based QA analysis. %GP for individual volumes of PTV (*γ*PTV) and GTV (*γ*GTV) with 2%/2 mm criteria were less than 90% for both NPC and prostate cancer patients. All other individual volume gamma indices were all with acceptable %GPs (> 90%) for both model‐based and measurement‐based QA. Global %GPs were all acceptable for model‐based and measurement‐based QA. The global %GPs of prostate cancer patients were relatively higher than those of NPC with 0.98 ± 0.01, 0.96 ± 0.02, 0.99 ± 0.04, and 0.99 ± 0.003, 0.99 ± 0.004, 1.00 ± 0.00 for model‐based and measurement‐based QA of 2%/2 mm, 3%/3 mm, and 4%/4 mm criteria, respectively.

**Table 2:** Percentage gamma pass rates of individual volume gamma and global gamma for NPC and prostate cancer patients

	2%/2 mm		3%/3 mm		4%/4 mm	
	Model based	Measurement	Model based	Measurement	Model based	Measurement
NPC						
*γ*_GTV_	0.81 ± 0.10	0.83 ± 0.07	0.95 ± 0.04	0.95 ± 0.05	0.99 ± 0.01	0.99±0.01
*γ*_PTV_	0.82 ± 0.06	0.84 ± 0.04	0.95 ± 0.03	0.95 ± 0.03	0.99 ± 0.01	0.99 ± 0.01
*γ*_Brainstem_	0.96 ± 0.04	0.92 ± 0.10	0.99 ± 0.01	0.99 ± 0.03	0.99 ± 0.00	0.99 ± 0.006
*γ*_Cord_	0.92 ± 0.07	0.95 ± 0.05	0.99 ± 0.03	0.99 ± 0.03	0.99 ± 0.01	0.99 ± 0.01
*γ*_Left parotid_	0.96 ± 0.03	0.96 ± 0.04	0.99 ± 0.01	0.99 ± 0.01	0.99 ± 0.002	0.99 ± 0.001
*γ*_Right parotid_	0.97 ± 0.03	0.94 ± 0.05	0.99 ± 0.003	0.99 ± 0.008	0.99 ± 0.001	0.99 ± 0.001
*γ*_Lens_	0.89 ± 0.17	0.95 ± 0.09	0.99 ± 0.04	0.97 ± 0.02	0.99 ± 0.001	1.00 ± 0.00
Global gamma	0.94 ± 0.02	0.92 ± 0.03	0.98 ± 0.02	0.97 ± 0.02	0.99 ± 0.01	0.98 ± 0.02
Prostate cancer						
*γ*_GTV_	0.83 ± 0.12	0.78 ± 0.16	0.95 ± 0.04	0.93 ± 0.06	0.99 ± 0.02	0.99 ± 0.02
*γ*_PTV_	0.83 ± 0.12	0.77 ± 0.15	0.96 ± 0.05	0.93 ± 0.06	0.99 ± 0.02	0.99 ± 0.02
*γ*_Left femoral head_	0.98 ± 0.02	0.95 ± 0.06	0.99 ± 0.002	0.99 ± 0.004	0.99 ± 0.00	0.99 ± 0.006
*γ*_Right femoral head_	0.99 ± 0.02	0.97 ± 0.05	0.99 ± 0.03	0.99 ± 0.03	0.99 ± 0.01	0.99 ± 0.01
*γ*_Bladder_	0.97 ± 0.03	0.96 ± 0.05	0.99 ± 0.01	0.99 ± 0.01	0.99 ± 0.002	0.99 ± 0.001
*γ*_Rectum_	0.94 ± 0.06	0.91 ± 0.06	0.99 ± 0.003	0.98 ± 0.03	0.98 ± 0.03	0.99 ± 0.001
Global gamma	0.98 ± 0.01	0.96 ± 0.02	0.99 ± 0.04	0.99 ± 0.003	0.99 ± 0.004	1.00 ± 0.00

Table 3 shows the correlations between metrics dose difference and individual volume %GP and global %GP with different gamma analysis criteria for NPC. %GPs of individual volumes had a higher percent of correlation with individual metrics dose differences compared with global %GPs. For individual volume gamma indices, 9, 12, and 9 out of 15 %DE metrics were correlated with individual volume %GP for 2%/2 mm, 3%/3 mm, and 4%/4 mm criteria, respectively. For global %GP, only 2 out of 15 %DE metrics were correlated with %GP for 2%/2 mm, 3%/3 mm, and 4%/4 mm criteria, respectively.

**Table 3:** Correlations between metrics dose difference and different percentage gamma rate for NPC

%GP	Individual volume 2%/2 mm		Individual volume 3%/3 mm		Individual volume 4%/4 mm		Global 2%/2 mm		Global 3%/3 mm		Global 4%/4 mm	
Metrics	r	p	r	p	r	p	r	p	r	p	r	p
GTV												
Dmean	0.64	<0.001	0.60	<0.001	0.50	<0.001	0.04	0.74	0.15	0.26	0.08	0.52
D2	0.48	<0.001	0.53	<0.001	0.31	0.02	0.13	0.33	0.22	0.09	0.19	0.14
D98	0.51	<0.001	0.42	0.001	0.45	<0.001	−0.13	0.31	−0.12	0.36	0.18	0.16
V95	0.40	0.001	0.32	0.01	0.41	0.001	−0.06	0.67	−0.02	0.85	−0.04	0.78
PTV												
Dmean	0.25	0.05	0.35	0.006	0.15	0.24	0.18	0.17	0.13	0.33	0.09	0.49
D2	0.17	0.18	0.30	0.02	0.20	0.12	0.12	0.34	0.24	0.06	0.20	0.13
D98	0.09	0.51	0.10	0.43	−0.13	0.31	0.08	0.54	0.03	0.81	0.01	0.94
V95	0.56	<0.001	0.59	<0.001	0.35	0.006	−0.17	0.18	−0.05	0.72	−0.04	0.78
Brainstem D1	0.43	<0.001	0.50	0.02	0.41	0.001	0.41	0.001	0.41	0.001	0.35	0.005
Cord D1	0.08	0.54	0.18	0.17	0.15	0.26	0.06	0.65	0.08	0.52	0.10	0.44
Left parotid												
Dmean	−0.54	<0.001	−0.6	<0.001	−0.43	0.001	−0.009	0.95	−0.03	0.81	−0.04	0.75
D50	−0.43	<0.001	−0.4	<0.001	−0.46	<0.001	−0.34	0.008	−0.36	0.004	−0.33	0.01
Right parotid												
Dmean	−0.04	0.75	−0.20	0.07	−0.11	0.40	0.19	0.14	0.02	0.86	−0.06	0.64
D50	−0.18	0.15	−0.30	0.03	0.20	0.12	0.11	0.42	−0.08	0.55	−0.14	0.26
Lens D1	−0.59	<0.001	−0.40	0.001	−0.34	0.007	−0.008	0.95	0.009	0.95	−9.02	0.90

The correlations between 15 %DE metrics and individual volume %GP and global %GP for prostate cancer patients were presented in Table 4. There were 18, 16, and 13 out of 23 metrics dose differences correlated with individual volume %GPs with 2%/2 mm, 3%/3 mm, and 4%/4 mm criteria, respectively. While, 19, 12, and 1 out 23 %DE metrics were correlated with global %GPs for 2%/2 mm, 3%/3 mm, and 4%/4 mm criteria, respectively.

**Table 4:** Correlations between metrics dose difference and different gamma analysis for prostate cancer patients

%GP	Individual volume 2%/2 mm		Individual volume 3%/3 mm		Individual volume 4%/4 mm		Global 2%/2 mm		Global 3%/3 mm		Global 4%/4 mm	
Metrics	r	p	r	p	r	p	r	p	r	p	r	p
GTV												
Dmean	0.94	<0.001	0.84	<0.001	0.66	<0.001	0.4	0.001	0.25	0.04	0.17	0.17
D2	0.71	<0.001	0.69	<0.001	0.59	<0.001	0.45	<0.001	0.27	0.03	0.14	0.28
D98	0.84	<0.001	0.75	<0.001	0.59	<0.001	0.48	<0.001	0.32	0.008	0.11	0.36
V95	0.53	<0.001	0.59	<0.001	0.58	<0.001	0.31	0.01	0.23	0.06	0.18	0.15
PTV												
Dmean	0.95	<0.001	0.86	<0.001	0.69	<0.001	0.43	<0.001	0.27	0.03	0.16	0.21
D2	0.70	<0.001	0.68	<0.001	0.60	<0.001	0.46	<0.001	0.28	0.02	0.13	0.32
D98	0.84	<0.001	0.75	<0.001	0.63	<0.001	0.52	<0.001	0.34	0.005	0.09	0.46
V95	0.76	<0.001	0.75	<0.001	0.67	<0.001	0.38	0.002	0.26	0.03	0.24	0.049
Left femoral head												
Dmean	0.56	<0.001	0.29	0.02	−0.17	0.17	0.60	<0.001	0.34	0.005	0.19	0.12
D2	0.40	0.001	0.14	0.27	−0.25	0.04	0.24	0.05	0.15	0.22	0.06	0.66
Right femoral head												
Dmean	0.41	0.001	0.15	0.23	0.20	0.12	0.28	0.02	0.11	0.37	0.05	0.70
D2	0.24	0.049	0.11	0.37	0.21	0.09	0.11	0.37	0.09	0.49	0.18	0.15
Bladder												
Dmean	0.30	0.01	0.32	0.009	0.24	0.06	0.49	<0.001	0.36	0.003	−0.10	0.48
D40	0.21	0.08	0.20	0.10	0.13	0.31	0.34	0.005	0.24	0.05	−0.13	0.3
D70	0.12	0.34	0.14	0.27	0.08	0.51	0.45	<0.001	0.43	<0.001	−0.02	0.85
V40	0.07	0.60	0.06	0.64	0.05	0.59	0.37	0.002	0.30	0.01	−0.17	0.18
V60	−0.01	0.93	0.02	0.89	0.06	0.65	0.18	0.14	0.10	0.43	−0.17	0.18
V70	0.20	0.12	0.16	0.21	0.10	0.45	0.30	0.02	0.13	0.29	−0.05	0.67
Rectum												
Dmean	0.72	<0.001	0.59	<0.001	0.49	<0.001	0.50	<0.001	0.27	0.03	0.23	0.06
D5	0.55	<0.001	0.50	<0.001	0.47	<0.001	0.25	0.046	0.09	0.47	0.15	0.22
V50	0.40	0.001	0.26	0.03	0.22	0.08	0.33	0.008	0.12	0.34	0.03	0.81
V60	0.34	0.006	0.25	0.047	0.27	0.03	0.31	0.01	0.16	0.19	0.18	0.14
V70	0.60	<0.001	0.46	<0.001	0.32	0.008	0.23	0.06	0.10	0.43	0.11	0.37

**Figure 2:**
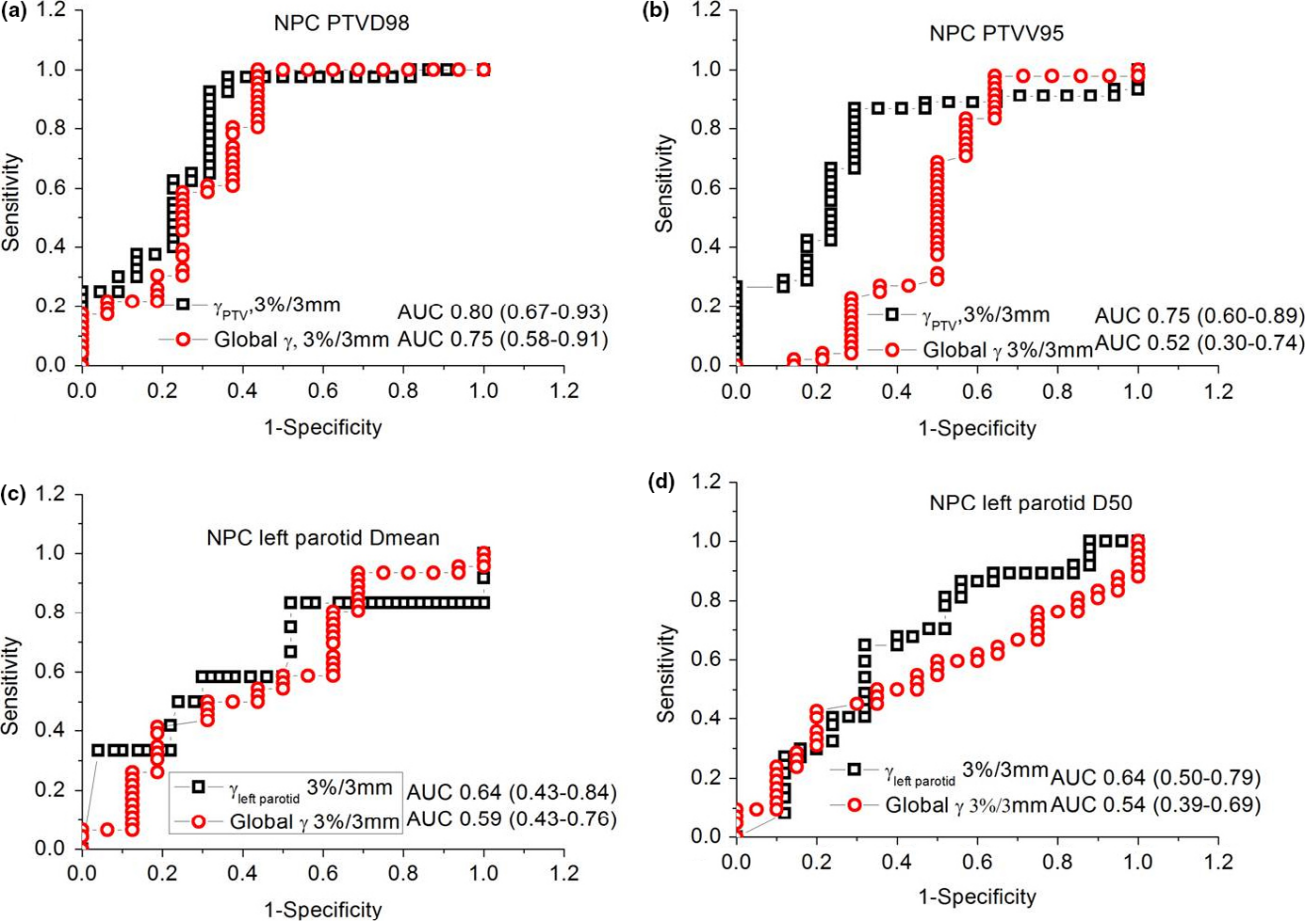
Comparative ROC curves between individual volume gamma indices and global gamma indices on different DVH metrics for NPC patients: (a) the D98 of PTV; (b) the V95 of PTV; (c) the Dmean of left parotid; (d) the D50 of left parotid.

**Figure 3:**
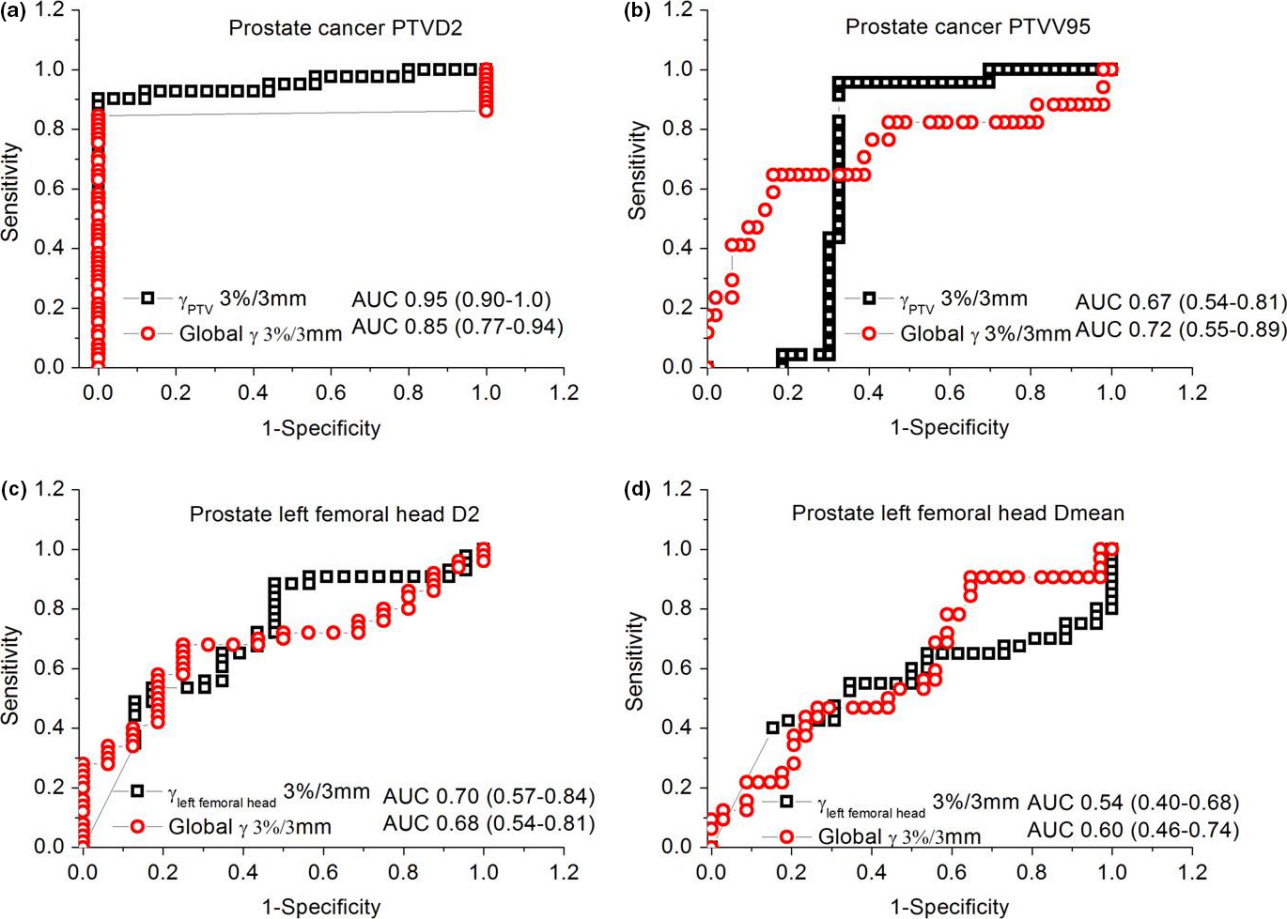
Comparative ROC curves between individual volume gamma indices and global gamma indices on different DVH metrics for prostate cancer patients: (a) the D2 of PTV; (b) the V95 of PTV; (c) the D2 of left femoral head; (d) the Dmean of left femoral head.

Further analysis on sensitivity was performed using the 3%/3 mm acceptance criterion, since 4%/4 mm showed less correlation and 2%/2 mm showed a lower passing rate. Figures 2 and 3 present the comparative ROC curves between individual volume %GPs and global %GPs on some of the DVH metrics for NPC and prostate cancer patients. The area under curves (AUC) of individual volume %GPs were higher than those of global %GPs for NPC patients. However, for prostate cancer patients, the AUC values were not straightforward between individual volume and global %GPs with mixed higher and lower results for different DVH metrics.

## 4 Discussion

The 3D individual volume %GP and global %GP, as well as their correlations with %DE of metrics between TPS‐ and QA‐reconstructed dose for both model‐based and measurement‐based VMAT QA were investigated on two‐arc VMAT NPC and one‐arc VMAT prostate cancer patients. Individual volume %GPs had a higher correlation with %DE of metrics for both two‐arc VMAT NPC and one‐arc VMAT prostate cancer patients compared with global %GPs. AUCs of individual volume %GPs were higher than those of global %GPs for NPC patients.

Due to the higher complexity of dose calculation in IMRT and VMAT treatment plans compared with 3D conformal radiotherapy (3D‐CRT), TPS performance verification and the independent verification of the dose calculation algorithm play an equally important role like verification of the treatment delivery and the comparison of the TPS‐calculated dose versus measured dose.^21^ Suggestions even had been made to switch from measurement‐based QA to model‐based QA to improve the time efficiency since research had shown that no dose delivery error occurred in 99.5% of the treatment plans.^22^ In this study, the VMAT dose calculation performed by Pinnacle TPS was verified with the COMPASS system using an inbuilt beam model as a model‐based QA process. The %DE of model‐based QA for most of DVH metrics of VMAT plans were within 3%, except for some metrics with sharp dose gradient (such as V95 and V70), and metrics with small volumes (lens). This is a bit different from reported IMRT QA with a second TPS, in which a very high agreement ratio (0.999) between initial and second TPS calculation were presented.^23^ However, only one point dose difference was reported in that study.

Although model‐based QA is time efficient and clinically feasible,^21^ IMRT and VMAT treatments also involve complex linac behaviors, such as correct data transfer, linac output, MLC leaf movement, gantry/collimator angles, etc. Measurement‐based QA using gamma analysis is still a standard practice for IMRT and VMAT. Similar to the reported lacking of correlation between %GP and clinical relevant metrics in previous IMRT and VMAT measurement‐based QA,^6^, ^24^ in this study, the correlations between global %GP and DVH metrics dose differences were very weak for two‐arc VMAT NPC (2 out of 15). The correlation between global %GP and DVH metrics dose differences for one‐arc VMAT prostate was better with 2%/2 mm criteria, but it was still very weak for clinical generally accepted gamma criteria 3%/3 mm (12 out of 23), and it was very weak for 4%/4 mm criteria (1 out of 23).

Due to this lack of correlation between global %GP and %DE, DVH metrics‐based QA comparing directly the TPS calculated and measured 3D dose distribution for IMRT and VMAT has been suggested.^6^, ^25^ The accuracy and feasibility of COMPASS 3D verification system for IMRT and VMAT plans have been investigated by many authors.^18^, ^26^, ^27^ A bit larger %DE between two algorithms on some metrics was observed, especially on metrics with shape gradient and small volumes. This could result from the intrinsic sensitivity discrepancy between different algorithms. However, a higher percent of correlation between individual volume %GP and 15 %DE metrics was observed for both two‐arc VMAT NPC patients and one‐arc VMAT prostate cancer patients in this study. This demonstrated the feasibility of utilizing direct prediction of the patient DVH and individual volume %GP instead of global %GP for pretreatment VMAT QA. Similarly, Wu et al. also concluded it was feasible to *γ*_PTV_ and *γ*_10%_ as 3D *γ*‐analysis quantities for IMRT and VMAT QA based on EPID dose back‐projection.^9^

Individual volume gamma indices were better predictors compared with global gamma indices for two‐arc VMAT NPC patients as shown by the ROC analysis of Fig. 2. The ROC analysis on prostate cancer patients indicated the individual volume %GPs were not very sensitive as they did for NPC patients. This could be due to the relative small target volumes and less complexity of one‐arc VMAT plans of prostate compared with NPC. We probably need a more strict acceptance criteria for one‐arc VMAT plans in our future study. This is also one of the limitations of this study, currently, we still could not figure out which individual DVH metrics was the most sensitive index for two‐arc and one‐arc VMAT QA. A more comprehensive review of the QA results beyond gamma analysis should be done, such as additional representative point dose check, isodose overlay check in three planes, DVH, and dose statistics checks for all PTVs and critical structures, etc.

## 5 Conclusion

In this study, the feasibility of 3D individual volume *γ‐*analysis was investigated for both model‐based and measurement‐based VMAT QA with COMPASS 3D verification system. Individual volume‐based 3D %GP had a higher percent of correlation with DVH‐based 15 %DE metrics compared with global %GP for both two‐arc VMAT NPC and one‐arc VMAT prostate cancer patients, indicating the sensitivity of using individual volume‐based 3D gamma indices for VMAT QA.

## Conflicts of interest

The authors declare no conflict of interest.
